# Neuroprotection by the Immunomodulatory Drug Pomalidomide in the *Drosophila* LRRK2^WD40^ Genetic Model of Parkinson’s Disease

**DOI:** 10.3389/fnagi.2020.00031

**Published:** 2020-02-13

**Authors:** Maria Antonietta Casu, Ignazia Mocci, Raffaella Isola, Augusta Pisanu, Laura Boi, Giovanna Mulas, Nigel H. Greig, Maria Dolores Setzu, Anna R. Carta

**Affiliations:** ^1^CNR Institute of Translational Pharmacology, Cagliari, Italy; ^2^Department of Biomedical Sciences, University of Cagliari, Cagliari, Italy; ^3^CNR Institute of Neuroscience, Cagliari, Italy; ^4^Department of Life and Environmental Sciences, University of Cagliari, Cagliari, Italy; ^5^National Institute of Aging (NIA), Drug Design & Development Section, Translational Gerontology Branch, Baltimore, MD, United States

**Keywords:** Parkinson, *Drosophila*, LRRK2, immunomodulation, neuroprotection, drug repurposing, repositioning

## Abstract

The search for new disease-modifying drugs for Parkinson’s disease (PD) is a slow and highly expensive process, and the repurposing of drugs already approved for different medical indications is becoming a compelling alternative option for researchers. Genetic variables represent a predisposing factor to the disease and mutations in leucine-rich repeat kinase 2 (LRRK2) locus have been correlated to late-onset autosomal-dominant PD. The common fruit fly *Drosophila* melanogaster carrying the mutation LRRK2 loss-of-function in the WD40 domain (LRRK2^WD40^), is a simple *in vivo* model of PD and is a valid tool to first evaluate novel therapeutic approaches to the disease. Recent studies have suggested a neuroprotective activity of immunomodulatory agents in PD models. Here the immunomodulatory drug Pomalidomide (POM), a Thalidomide derivative, was examined in the *Drosophila* LRRK2^WD40^ genetic model of PD. Mutant and wild type flies received increasing POM doses (1, 0.5, 0.25 mM) through their diet from day 1 post eclosion, until postnatal day (PN) 7 or 14, when POM’s actions were evaluated by quantifying changes in climbing behavior as a measure of motor performance, the number of brain dopaminergic neurons and T-bars, mitochondria integrity. LRRK2^WD40^ flies displayed a spontaneous age-related impairment of climbing activity, and POM significantly and dose-dependently improved climbing performance both at PN 7 and PN 14. LRRK2^WD40^ fly motor disability was underpinned by a progressive loss of dopaminergic neurons in posterior clusters of the protocerebrum, which are involved in the control of locomotion, by a low number of T-bars density in the presynaptic bouton active zones. POM treatment fully rescued the cell loss in all posterior clusters at PN 7 and PN 14 and significantly increased the T-bars density. Moreover, several damaged mitochondria with dilated cristae were observed in LRRK2^WD40^ flies treated with vehicle but not following POM. This study demonstrates the neuroprotective activity of the immunomodulatory agent POM in a genetic model of PD. POM is an FDA-approved clinically available and well-tolerated drug used for the treatment of multiple myeloma. If further validated in mammalian models of PD, POM could rapidly be clinically tested in humans.

## Introduction

Parkinson’s disease (PD) is a progressive neurodegenerative disorder whose pathology primarily targets dopaminergic neurons of the substantia nigra pars compacta, together with non-motor areas of the CNS. This leads to typical PD-associated motor symptoms, which includes bradykinesia, tremor, and rigidity, as well as a range of non-motor symptoms, typified by postural instability, cognitive impairment and olfactory deficits (Parkinson, 2002; Dauer and Przedborski, 2003; Lees et al., 2009; Chaudhuri and Odin, 2010; Erkkinen et al., 2018). The degeneration of dopamine neurons in the substantia nigra is progressive and motor symptoms appear predictably when cell loss exceeds 50 percent.

The development of new drugs for the treatment of PD has been a slow and highly expensive process and, to date, has led to the approval of drugs that have proved to be symptomatic in their efficacy, but ineffective in slowing disease progression. As a response to these limitations and in the light of increasing knowledge of PD neuropathology, the pre-clinical and clinical testing of drugs with regulatory-approved clinical safety data, namely drug-repurposing, has become a compelling option for the pharmaceutical industry and researchers in the search for new PD therapeutics. Drug repurposing or repositioning is a strategy aimed at identifying new uses for already approved or investigational drugs, that fall outside their original medical indication (Brundin et al., 2013; Pushpakom et al., 2019).

Neuroinflammatory mechanisms are recognized as contributors to the neuropathology of PD and are classically characterized by reactive microgliosis with excessive production of proinflammatory molecules within degenerating mesencephalic areas (Troncoso-Escudero et al., 2018). Under physiological conditions, neuroinflammatory responses are finely orchestrated *via* a regulated production of cytokines, growth factors and toxic free radicals. On the contrary, in the case of PD, deregulation of neuroinflammatory responses occurs, and the chronic release of inflammatory cytokines, such as tumor necrosis factor (TNF)-α, is regarded as a principal pathological contributor to the ensuing progressive neurodegeneration (Joers et al., 2017). In this light, pharmacologically targeting the mechanisms underpinning cytokine production or actions may provide a compelling disease-modifying strategy for PD (Martinez and Peplow, 2018).

Based on the recognized role of neuroinflammation in PD neuropathology, evaluating commercially available immunomodulatory drugs for repositioning in PD has been considered an auspicious approach (Martinez and Peplow, 2018). Different classes of clinically available drugs active on the immune system have been investigated across various experimental models of PD, suggesting a benefit in slowing the disease progression and the development of motor symptoms (Van der Perren et al., 2015; Ren et al., 2017; Zhao et al., 2017). In recent years, immunomodulatory drugs, such as Thalidomide and its derivatives Lenalidomide and Pomalidomide (POM), have been appraised for the treatment of neurological disorders with a neuroinflammatory component; however, their potential utility in PD models has, to date, been poorly investigated (Tweedie et al., 2011). Thalidomide and derivatives display a potent biological effect on cytokine-mediated responses, acting primarily through the inhibition of TNF-α production *via* posttranslational mechanisms, with consequent dampening of the inflammatory cascade (Sampaio et al., 1991; Moreira et al., 1993; Tweedie et al., 2011; Chanan-Khan et al., 2013; Terpos et al., 2013). Among Thalidomide-derived immunomodulatory compounds, POM holds particular interest for its potent anti-TNF-α activity at significantly lower concentrations than the parent compound, as described in embryos and *in vitro* assays (Mahony et al., 2013). Moreover, POM displayed less adverse effects than Thalidomide and Lenalidomide, in relation to its teratogenic, anti-angiogenic and neurotoxic activity (Mahony et al., 2013; Vargesson et al., 2013).

Although the majority of human PD cases are idiopathic, genetic variables may represent a predisposing factor to the disease, and mutations in several specific genes have been linked to familial forms of PD. Among them, multiple mutations in the leucine-rich repeat kinase 2 (LRRK2) gene have been correlated to late-onset autosomal dominant PD (Kumari and Tan, 2009; Hernandez et al., 2016), accounting for up to 13% of familial PD cases and have been detected in 1–2% of idiopathic PD cases, making LRRK2 the most commonly linked PD gene. LRRK2 holds a dual enzymatic activity with two domains involved, namely the N-terminal and the C-terminal WD40 domain (Mills et al., 2012). In particular, the missense substitution G2385R within the WD40 domain leads to a partial loss-of-function of LRRK2, and is pathologically relevant for PD, being associated with an increased risk of developing idiopathic PD in Chinese and Korean ethnicity (Tan et al., 2009; Carrion et al., 2017).

The common fruit fly *Drosophila* melanogaster (Dm) is a useful organism for modeling neurodegenerative diseases with a translational value, carrying 75% homology with human disease genes (Bilen and Bonini, 2005). Dm carrying the LRRK2 loss-of-function mutation in the WD40 domain (LRRK^WD40^) is a simple *in vivo* model of PD that recapitulates key features of the disease, including motor impairment and mitochondrial abnormalities (Lee et al., 2012; De Rose et al., 2016; Hewitt and Whitworth, 2017). Of note, signaling pathways that regulate immune responses are highly conserved throughout evolution (Buchmann, 2014) and cytokines are important mediators of the innate immune system used by *Drosophila* to fight pathogen invasion (Vanha-Aho et al., 2016). Specifically, *Drosophila* has the ortholog of the tumor necrosis factor (TNF) Eiger as well as of TNF receptor (TNFR) Wengen (Moreno et al., 2002; Igaki and Miura, 2014). Genetic studies have identified a high homology of Eiger with the human gene, and evolutionarily conserved functions (Moreno et al., 2002; Salazar-Jaramillo et al., 2014). Therefore, the *Drosophila* is a useful and simple model for targeting cytokines with therapeutic agents.

Here, we first characterized the progressive dopaminergic neuronal loss in the *Drosophila* LRRK2^WD40^ genetic model of PD. Thereafter, we investigated the neuroprotective outcome of the immunomodulatory agent POM in this model. Specifically, POM activity was evaluated across ages in fly motor performance and loss of dopamine neurons in anatomically identified cell clusters within the brain. Ultrastructural analysis of mitochondrial integrity, and of T-bars as the essential part of the molecular machinery mediating neurotransmitter release in *Drosophila*, was also performed (Wagh et al., 2006).

## Materials and Methods

Adult wild type (WT; Oregon-R) and LRRK^WD40^ mutants (LRRK^ex1^, #34750, from Bloomington Stock Center) Dm males were used. After emergence from pupae, WT or LRRK^WD40^ mutant males were separated from females, reared and tested at ages 1–7–14 days (respectively PN 1, PN 7, PN 14). WT and mutant flies were reared on a standard cornmeal-yeast-agar medium in controlled environmental conditions (24–25°C; 60% relative humidity; light/dark = 12/12 h). In addition, groups of mutant and WT flies were reared on the standard medium supplemented with increasing concentrations of POM (0, 25–0, 5–1–2 mM) for 1 and 2 weeks.

### Climbing Assay

The climbing assay (negative geotaxis assay) was used to assess locomotor ability (Liu et al., 2008; De Rose et al., 2016). Climbing data were obtained from different age groups (PN 1, PN 7 and PN 14) of untreated and treated WT and LRRK2^WD40^ mutants. Cohorts of 60 flies from each experimental group were subjected to the assay. Flies were placed individually in a vertically-positioned plastic tube (length 10 cm; diameter 1.5 cm) and tapped to the bottom. Climbing time (s) was recorded upon crossing a line drawn at 6 cm from the bottom. The number of flies that could climb onto, or above this line within 10 s was recorded and expressed as a percentage of total flies evaluated. Data were expressed as the average ± standard error of the mean (SEM) from three different experiments.

### Immunohistochemistry

Free-floating fluorescent immunostaining for Tyrosine Hydroxylase (TH) was performed on the whole dissected adult brain from 6 to 8 flies from each experimental group.

LRRK2^WD40^ and WT *Drosophila* were anesthetized on ice, and brains were rapidly dissected and fixed in 4% paraformaldehyde in phosphate-buffered saline (PBS). After fixation and washes in PBS, whole brains were incubated with the TH primary antibody (1:100; AB 152 Millipore) and 10% normal donkey serum in PBS + 0.3% Tween 20 (PBST), at 4°C for 72 h. After washing, the brains were incubated with a donkey anti-rabbit Alexa Fluor 594 secondary antibody (1:200 Jackson ImmunoResearch, West Grove, PA, USA) and 10% normal donkey serum in PBST at 4°C for another 72 h. Afterward, the brains were mounted on glass slides and coverslipped with Vectashield.

Fluorescent images were captured with a fluorescence spinning disk confocal microscope (Crisel Instruments). The brains were scanned through Z-stacks (63× objective) and the number of TH-positive neurons was calculated in identified cell clusters, including paired posterior lateral clusters PPL1, PPL2, paired posterior medial clusters PPM1/2, PPM3, paired anterior lateral and medial clusters PAL and PAM. Cells were counted manually in both hemispheres for each stack *via* NIH ImageJ software, by an observer blinded to the treatments.

### Electron Microscopy Analysis

LRRK2^WD40^ mutants untreated or treated with POM 0.5 mM (*n* = 3) were anesthetized on ice, and brains were rapidly dissected and fixed in a mixture of 1% paraformaldehyde and 1.25% glutaraldehyde in 0.15 M cacodylate buffer. After fixation and rinsing in the same buffer, brains were post-fixed with 1% osmium tetroxide in distilled water for 2 h and then stained overnight with 0.5% uranyl acetate at 4°C. Brains were then dehydrated in a graded acetone series, and embedded in EPON resin. To identify the protocerebrum, semi-thin coronal sections of the whole brains, cut with a Reichert Supernova ultramicrotome, were stained with toluidine blue. Ultrathin sections (90 nm) were observed under a JEOL JEM 1400 Plus electron microscope, equipped with a CCD camera, at an acceleration voltage of 80 kV.

Morphometry: the total number of mitochondria, the percentage of mitochondria with swollen cristae (expressed as the percentage of mitochondria displaying swollen cristae out of total mitochondria with discernible cristae in the sampled area) and the T-bars density was evaluated in the unitary area (25 μm^2^) within dorsal or ventral protocerebrum, in each experimental group. Forty to fifty unitary area fields for each area were considered for each brain. More than 10,970 mitochondria and 1,400 T-bars were randomly sampled on 504 non-overlapping micrographs at a final magnification of 10,000×. Swollen cristae were recognized when the individual cristae size (i.e., the distance between two contiguous membranes of one crista) doubled the average cristae size. T-bars were unambiguously identified at presynaptic active zones by the presence of T-shaped electron-dense projections.

### Statistics

Statistically significant differences between groups in the behavioral and immunohistochemical analysis were evaluated by one-way or two-way ANOVA followed by Tukey’s multiple comparisons test when appropriate. TEM analysis was performed Mann–Whitney Rank Sum Test as data did not follow a normal distribution. A *P*-value ≤ 0.05 was considered significant.

## Results

### LRRK2^WD40^ Mutants Display Cardinal Features of PD

We first evaluated LRRK2^WD40^ mutants, as compared to WT Oregon-R flies of increasing ages (PN 1–7–14), for motor impairment and loss of dopaminergic neurons as these are two cardinal features of PD.

**Figure 1:**
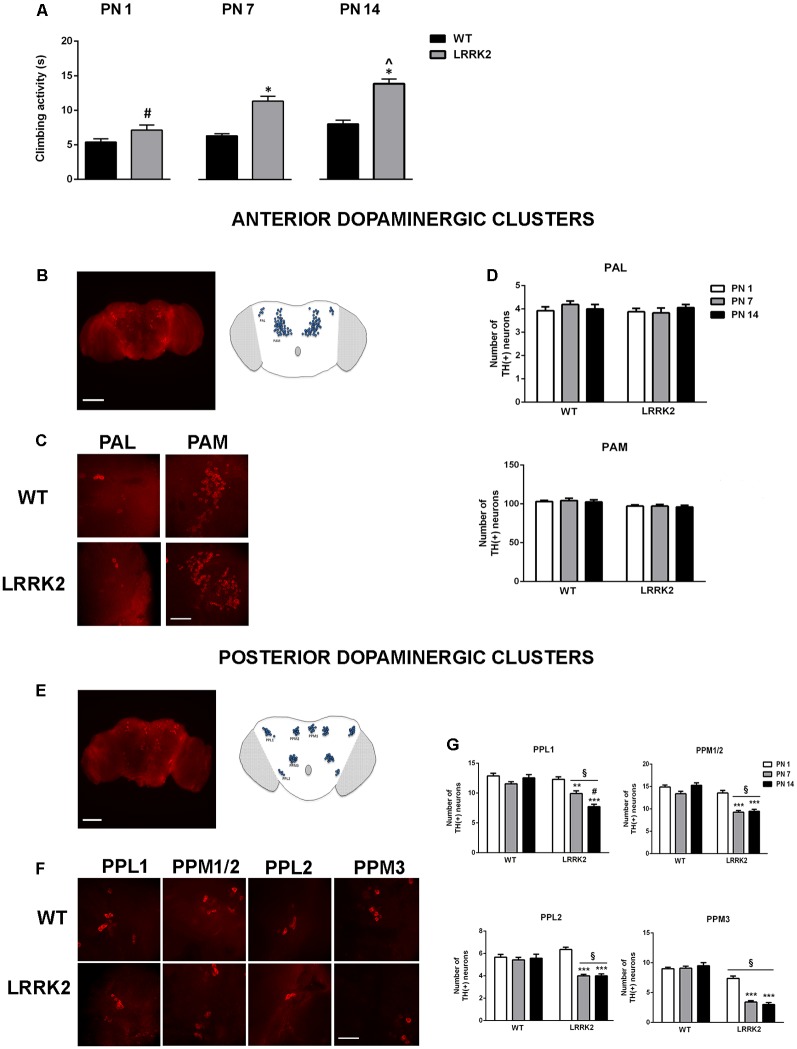
**(A)** Climbing behavior of wild type (WT) and leucine-rich repeat kinase 2 (LRRK2)^WT40^ (LRRK2) flies at postnatal days (PN) 1, 7, 14. Bars represent the mean ± SEM of 60 flies per group. *p < 0.001 vs. WT, ^#^*p* < 0.001 vs. LRRK2 at PN 7 and PN 14 days, respectively; ^∧^*p* < 0.05 vs. LRRK2 PN 7, by two-way ANOVA followed by Tukey’s *post hoc*. **(B,C)** Representative images (10×) of the anterior brain **(B)** and higher magnification (63×) of anterior dopaminergic clusters PAM and PAL **(C)** of the *Drosophila* brain. **(D)** Number of dopaminergic neurons in the anterior dopaminergic clusters PAM and PAL in WT and LRRK2 mutants at PN 1, 7, 14. **(E–F)** Representative images (10×) of the posterior brain **(E)** and higher magnification (63×) of posterior dopaminergic clusters PPL1, PPL2, PPM1/2, PPM3 **(F)** of the *Drosophila* brain. **(G)** Number of dopaminergic neurons in the posterior dopaminergic clusters in WT and LRRK2 mutants at PN 1, 7, 14. ^§^*p* < 0.05 vs. the corresponding WT; **,***p < 0.05 and 0.01 vs. LRRK2 PN 1; ^#^*p* < 0.05 vs. PN7, by two-way ANOVA followed by Tukey’s *post hoc*.

The climbing assay (negative geotaxis assay) was used to assess locomotor ability. The analysis of climbing activity showed that LRRK2^WD40^ mutants developed an age-related motor impairment measured as the time spent to reach the target. At PN 1 LRRK2^WD40^ displayed a climbing activity similar to WT flies, while at PN 7 and PN 14 LRRK2^WD40^ mutants displayed a significant motor impairment as compared to WT flies, as shown by the strain effect (*F*_(1,472)_ = 59.64, *P* < 0.0001), age effects (*F*_(2,472)_ = 24.80, *P* < 0.0001) and strain × age interaction (*F*_(2,472)_ = 5.271, *P* < 0.01; Figure 1A). Moreover, the motor impairment was significantly greater at PN 14 than PN 7, suggesting a progressive deterioration of motor abilities (*P* < 0.05 as compared to PN14 by Tukey’s *post hoc* test; Figure 1A).

We then evaluated the integrity of dopaminergic neurons in anterior and posterior neuronal clusters of the *Drosophila* brain *via* analysis of TH IR (Figures 1B–G). The number of anterior dopamine cell clusters proved to be stable across both WT flies and LRRK2^WD40^ mutants across all ages analyzed (Figures 1B–D). In contrast, dopaminergic neurons of posterior clusters underwent an age-related reduction in LRRK2^WD40^ but not in WT flies (*P* < 0.01 as compared to PN 1 by Tukey’s *post hoc* test), in accord with the role played by these nuclei in *Drosophila* motor activity (Figures 1E–G). Of note, the loss of dopaminergic neurons in PPL1 was gradual, with a lower number of neurons at PN 14 as compared with PN 7 (*P* < 0.05 vs. PN 7), in line with the progressive deterioration of motor ability (Figure 1E). Interestingly, LRRK2^WD40^ flies showed a significantly lower number of dopaminergic neurons within PPM3 as compared with WT flies, already at PN 1 (Figure 1G).

### POM Prevented the Age-Dependent Motor Impairment in LRRK2^WD40^ *Drosophila*

**Figure 2:**
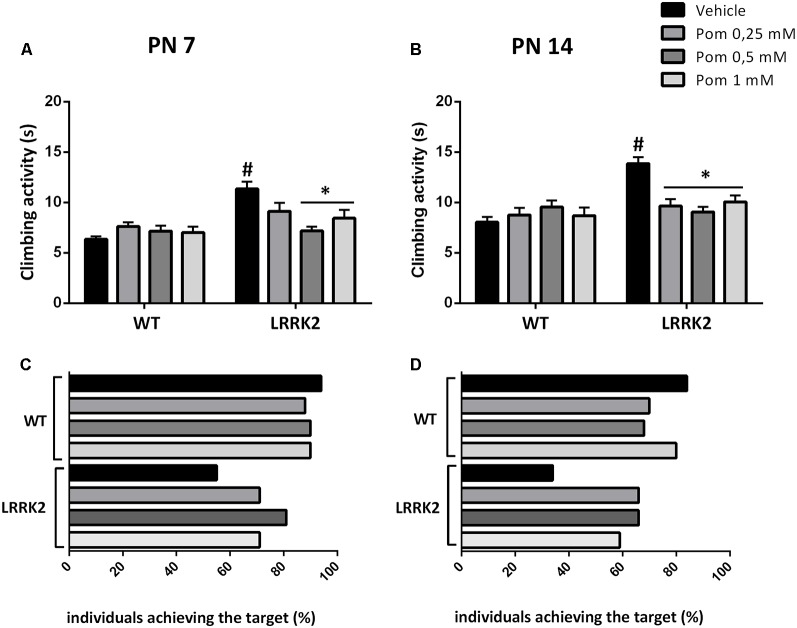
**(A,B)** Climbing activity of WT and LRRK2 mutant flies treated with increasing concentrations of pomalidomide (POM) in their diet at PN 7 **(A)** and 14 **(B)**. **(C,D)** Percentage of WT and LRRK2 individuals reaching the target within 10 s. ^#^*p* < 0.01 vs. the corresponding WT; *p < 0.01 vs. vehicle, by two-way ANOVA followed by Tukey’s *post hoc*.

LRRK2^WD40^ and WT flies were exposed to increasing doses of POM in their diet (0.25–0.5–1 mM) starting from PN 1, to evaluate the effect on locomotor ability parameters. The motor test was performed at PN 7 and PN 14, when the climbing activity and the percentage of flies reaching the target within 10 s was measured. The exposure to 0.5 and 1 mM POM for 7 days (PN 7) reduced the climbing time in LRRK2^WD40^ as compared to vehicle-exposed mutants (strain effect: *F*_(1,628)_ = 19.97 *P* < 0.0001; treatment effect: *F*_(3,628)_ = 2.681 *P* < 0.05; strain × treatment interaction: *F*_(3,628)_ = 5.623, *P* < 0.001; Figure 2A), whereas the lowest POM concentration (0.25 mM) failed to produce any effect at this age. After 14 days of treatment (PN 14) a recovery of motor disability in the LRRK2^WD40^ was observed with all POM concentrations, as shown by the reduction of climbing time as compared to vehicle-exposed flies (strain: *F*_(1,560)_ = 14.93, *P* < 0.001; treatment: *F*_(3,560)_ = 2.892, *P* < 0.05; strain × treatment interaction: *F*_(3,560)_ = 7.783, *P* < 0.0001). The motor activity of WT flies was unaffected by POM at all tested concentrations (Figures 2A,B).

As an additional parameter of motor ability, we counted the percentage of WT and LRRK2^WD40^ flies completing the climbing test within 10 s (Figures 2C,D). We found that at PN 7, and even more so at PN 14, the percent of flies reaching the target was lower as compared to WT flies of the same age (respectively, 55% and 34% as compared to 94% in WT; Figures 2C,D). Moreover, exposure to all concentrations of POM for 7 or 14 days increased the number of LRRK2^WD40^ flies that reached the target at PN 7 and PN 14 (Figures 2C,D).

### POM Prevented the Neuronal Loss in LRRK2^WD40^*Drosophila*

**Figure 3:**
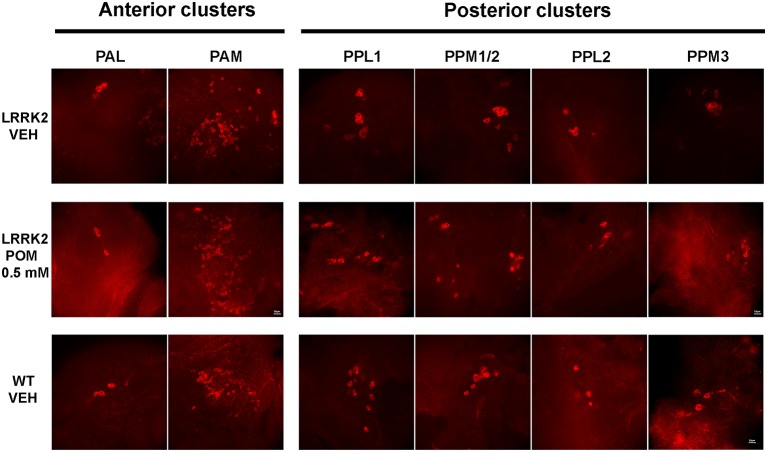
Representative images (63×) of tyrosine hydroxylase (TH)-positive neurons in anterior (PAL, PAM) and posterior (PPLs, PPMs) dopaminergic clusters, in WT and LRRK2 flies treated with vehicle or with POM 0.5 mM for 14 days (PN 14).

**Figure 4:**
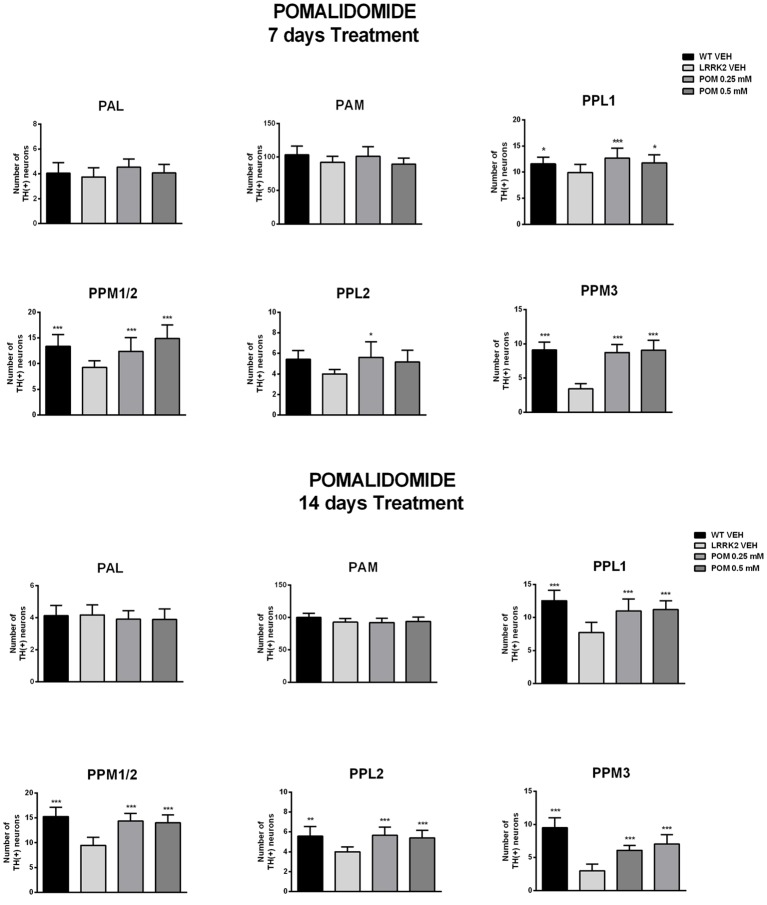
POM effect on the number of TH-positive neurons in anterior (PAM, PAM) and posterior (PPLs and PPMs) dopaminergic cluster of the *Drosophila* brain, from flies treated with vehicle, 0.25, 0.5 mM POM, after 7 and 14 days of treatment (PN 7 and PN 14). *,***p < 0.05 or 0.01 vs. LRRK2 VEH, by Two-way ANOVA followed by Tukey’s *post hoc*.

Flies were sacrificed after the climbing test to quantitatively evaluate POM’s actions on TH-IR in anterior and posterior brain clusters of dopaminergic neurons (Figures 3, 4). Since POM displayed a maximal behavioral effect at the concentration of 0.5 mM, with no further improvement at 1 mM, only brains from flies exposed to 0.25 and 0.5 mM were processed for TH immunohistochemistry.

The analysis of TH-IR confirmed that neuronal loss was confined to posterior clusters in LRRK2^WD40^ mutants and showed that treatment with POM prevented it. Specifically, exposure to POM 0.25 and 0.5 mM similarly increased the number of TH-positive neurons within the posterior clusters of the brain in LRRK2^WD40^ flies, namely PPL1, PPL2, PPM1/2 and PPM3 (Figure 4). Importantly, POM fully prevented the loss of dopamine neurons at PN 7 as well as at PN 14, when the neuronal loss in LRRK2^WD40^ was greater, in accord with the complete prevention of motor deficits. Exposure to POM did not produce any effect on TH-IR in WT flies (data not shown).

### POM Prevented the Loss of T-bars and Mitochondrial Damage in LRRK2^WD40^ *Drosophila*

**Figure 5:**
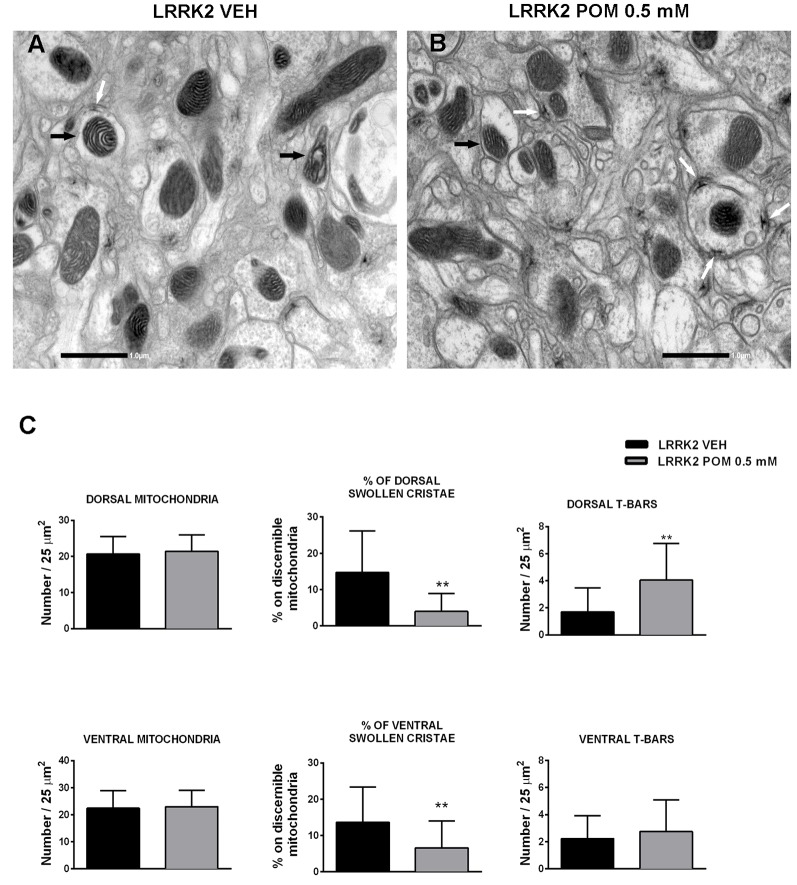
Representative images of mitochondria (10,000×) acquired from the protocerebrum of LRRK2 *Drosophila* treated with vehicle **(A)** or with POM 0.5 mM **(B)**. Black arrows indicate mitochondria with swollen cristae in **(A)** and with normal cristae in **(B)**; white arrows indicate T-Bars. **(C)** POM effect on the number of total mitochondria, percentage of swollen cristae and T-Bars in the dorsal and ventral protocerebrum. **p < 0.01 vs. LRRK2 VEH, by Mann–Whitney test.

Transmission electron microscopy (TEM) analysis was conducted on LRRK2^WD40^ flies treated with vehicle or with 0.5 mM POM for 14 days. Ultrastructural morphology of *Drosophila* LRRK2^WD40^ brain was investigated both in the dorsal and ventral protocerebrum and showed longitudinally cut axons and terminal boutons with numerous synaptic vesicles and T-bar shaped presynaptic densities (Figures 5A,B). The morphometric analysis did not reveal any difference in the number of total mitochondria between control and treated LRRK2^WD40^ (Figure 5C). Inside axons and terminal boutons of vehicle-treated LRRK2^WD40^ flies several pleomorphic mitochondria were present, some displaying swollen cristae (Figure 5A). POM treatment clearly decreased the occurrence of mitochondria with swollen cristae as compared with vehicle treatment, both in dorsal and ventral protocerebrum (*p* < 0.01; Figures 5B,C). Moreover, POM significantly increased the number of T-bar in the presynaptic bouton active zones of the dorsal protocerebrum as compared to vehicle treatment (*p* < 0.01; Figure 5C). Exposure of WT flies to POM did not produce any effect on mitochondria number and morphology, nor in the number of T-bars (data not shown).

## Discussion

In the present study, we investigated the neuroprotective activity of the immunomodulatory drug POM in a transgenic *Drosophila* model of PD. We demonstrate that POM effectively prevented motor deficits, protected against the progressive loss of dopaminergic neurons, increased the number of T-bars and reduced mitochondrial damage. To our knowledge, this is the first report evaluating POM as a neuroprotective agent in a PD model. Moreover, this is the first report on the efficacy of an immunomodulatory drug in a transgenic model of PD, which complement studies investigating the neuroprotective efficacy of these agents in toxin-based or alpha-synuclein-based models (Martinez and Peplow, 2018).

Based on the recognized role of neuroinflammation and inflammatory cytokines in the neuropathology of PD, clinically available agents that act on the central immune system *via* an immunosuppressive or immunomodulatory mechanism have been tested in experimental models of PD (Martinez and Peplow, 2018). Among drugs in clinical use, the immunosuppressants Fingolimod (Gilenya), Tacrolimus (Fujimycin) and Cyclosporin have been reported as effective neuroprotective treatments in rodent models of dopaminergic degeneration (Tamburrino et al., 2015; Van der Perren et al., 2015; Vidal-Martínez et al., 2016; Ren et al., 2017; Zhao et al., 2017). However, the systemic adverse effects of immunosuppressants and their narrow therapeutic window, together with their limited ability to cross the blood-brain barrier (BBB) heavily limit the use of these drugs in PD. Dimethyl Fumarate, another FDA-approved drug with a mixed mechanism of action that includes an anti-inflammatory activity, was reported effective in ameliorating PD features in an alpha-synuclein based model of PD (Lastres-Becker et al., 2016). More recently, the focus of research for the treatment of neurological disorders has moved toward the investigation of agents acting through an immunomodulatory, rather than immunosuppressive mechanism (Martinez and Peplow, 2018). Hence, given the dual beneficial and potentially harmful roles of activated immune cells within the CNS, the strategy of boosting reparative and protective functions, while suppressing pro-inflammatory activity and the detrimental effects of inflammatory cytokines, might be efficacious to achieve neuroprotection in PD (Frankola et al., 2011; Lecca et al., 2018). Thalidomide is a small molecule drug that acts by inhibiting the synthesis of the potent inflammatory cytokine TNF-α causing a consequent dampening of the inflammatory cascade. Unlike larger molecules, it is readily capable of crossing the BBB (Tweedie et al., 2007; Boi et al., 2019). Therefore, Thalidomide and its analogs are promising candidate agents for evaluation in a variety of neurological disorders underpinned by neuroinflammation (Tweedie et al., 2007, 2012; Yoon et al., 2013; Wang et al., 2016; Batsaikhan et al., 2019; Tsai et al., 2019). In PD models, Thalidomide and the analog Lenalidomide, administered at the same dose of 100 mg/kg p.o. to mice overexpressing alpha-synuclein, induced an improvement in motor performance and dopaminergic fibers loss (Valera et al., 2015). Regrettably, both drugs are associated with unwanted side effects when used in chronic regimens. Thalidomide is currently used mainly to treat multiple myeloma and erythema nodosum leprosum (Franks et al., 2004), but its long-term use at doses required to stop inflammation has been associated with peripheral neuropathy as well as teratogenic effects (Vargesson, 2009). Indeed, in a recent clinical trial of Thalidomide in Alzheimer’s disease, poor tolerability precluded the agent being administered at the perceived efficacious dose (Decourt et al., 2017). Lenalidomide is a more potent anti-inflammatory drug as compared with Thalidomide, effectively inhibiting TNF-α at lower doses than the parent compound. However, the drug has been reported to be a substrate for the p-glycoprotein efflux pump at the BBB, which may limit it achieving and maintaining therapeutic levels in the brain (Hughes et al., 2019). Furthermore, long-term use of Lenalidomide can also result in peripheral neuropathy (Christian et al., 2007; Palumbo et al., 2012; Voorhees et al., 2013) and, similar to Thalidomide, it displays teratogenic, antiangiogenic, and neurotoxic activity at anti-inflammatory doses (Mahony et al., 2013). Hence, Thalidomide derivatives with potent anti-inflammatory properties but fewer side effects are currently under investigation (Beedie et al., 2016; Luo et al., 2018). Among them, POM is an FDA-approved and clinically available immunomodulatory drug in use for the treatment of multiple myeloma that is resistant to Lenalidomide (Siegel et al., 2019). POM is a structural analog of Thalidomide, with potent anti-inflammatory activity at significantly lower concentrations than the parent compound or Lenalidomide, and with a TNF-α inhibitory action of up to 50,000-fold greater than Thalidomide (Mahony et al., 2013; Wang et al., 2016). In a recent study, POM displayed an excellent BBB permeability in mice, achieving a brain/plasma concentration ratio of 0.71 (Tsai et al., 2019). Moreover, unlike Thalidomide and Lenalidomide, POM was devoid of anti-angiogenic and teratogenic actions at potently anti-inflammatory concentrations (Mahony et al., 2013), suggesting that it may be a more effective and safer drug, which justifies its preferential use with respect to its analog compounds in cancer treatment (Shortt et al., 2013; Mo and Richardson, 2019). In neurological diseases, POM was proven effective in reducing neuronal loss in a rat model of moderate traumatic brain injury (Wang et al., 2016) and mitigated H2O2-induced death of cortical neurons in culture (Tsai et al., 2018) and neuronal loss in rats subjected to ischemic stroke (Tsai et al., 2019) *via* its anti-oxidative and anti-inflammatory activity; however, this drug has yet to be tested in PD.

In the present study, POM was tested in the *Drosophila* LRRK2 model of PD carrying the G2385R mutation within the WD40 protein domain. Several studies have demonstrated that the G2385R mutation is a pathologically relevant variant, causing a partial loss of the kinase function of LRRK2 (Rudenko et al., 2012; Carrion et al., 2017). Accordingly, an increased risk of developing idiopathic PD in Chinese and Korean ethnicity holding this mutation has been widely reported (Tan et al., 2009; Xie et al., 2014). Whereas many studies have characterized LRRK2 gain-of-function models of PD in the rodent and insect (Liu et al., 2008), only one previous study has validated the *Drosophila* LRRK2^WD40^ as a model of PD (De Rose et al., 2016), showing that LRRK2^WD40^ mutants displayed features of PD including motor disability, reduced life span and mitochondrial damage (De Rose et al., 2016). Here, we further validated the *Drosophila* LRRK2^WD40^ as a PD model by conducting a detailed analysis of dopaminergic neuron clusters in the fly brain in an age-dependent manner. In the *Drosophila* brain, DA neurons are organized in distinct bilateral symmetric clusters comprising a stereotypical number of cells, with specific projections onto target regions that represent distinct functional areas (Monastirioti, 1999; Lima and Miesenbock, 2005). While PAL neurons mostly innervate the optic tubercle, PPL1 neurons innervate distinct regions of the mushroom bodies that are implicated with learning and memory (Zars, 2000). Moreover, PPM3 neurons innervate the central complex, which is a higher center for the control of locomotion (Strauss, 2002).

We demonstrate an age-related loss of dopaminergic neurons in LRRK2^WD40^ mutants but not in WT flies. Of note, the dopaminergic cell loss was limited to the posterior dopaminergic clusters, while sparing the anterior clusters, in line with the prominent role played by posterior clusters in the control of locomotion (Strauss, 2002; Mao and Davis, 2009). Moreover, we show that the cell loss was absent in LRRK2^WD40^ mutants at birth (1–2 days old flies), with the exception of the PPM3, while a significant decrease in the number of TH-positive neurons was observed in 7 days old mutants, becoming even greater in older flies. This supports the progressive nature of dopaminergic degeneration, adding important translational value to the LRRK2^WD40^ fly as a model of PD. Accordingly, motor disability paralleled dopaminergic degeneration in our study, being recorded in 7 and 14 days old mutants but not at birth (1–2 days old). The age-related decline of dopamine neurons was dependent upon the presence of the G2385R mutation, since the number of cells remained unvaried in WT flies with aging, in line with previous studies showing lack of age-related degeneration in the brain of WT *Drosophila* (White et al., 2010). Confirming previous findings, we also observed the presence of damaged mitochondria as indicated by multiple dilated cristae (De Rose et al., 2016). Of note, the G2385R variant has been associated with reduced LRRK2 binding to synaptic proteins and reduced neurotransmission, which may underpin defective T-bars in the LRRK2^ WD40^ *Drosophila* model (Carrion et al., 2017).

This further demonstration of dopaminergic degeneration in an LRRK2-loss of function model adds a piece to a yet puzzling issue, where the pathological role of loss of function variants in PD is still debated (Blauwendraat et al., 2018). Kinase inhibitors blocking LRRK2 gain of function are currently considered as potential treatments in PD, suggesting that further studies are warranted to define the molecular mechanisms underpinning the involvement of LKKR2 variants in PD.

We report the neuroprotective effect of POM in the LRRK2^WD40^ model. This neuroprotective effect was established by the POM-mediated improvement of motor performance, the rescue of TH-positive neurons and mitigation of mitochondrial damage. Moreover, POM recovered the loss of T-bars, the presynaptic active zone involved in neurotransmitter release in *Drosophila* (Fouquet et al., 2009). Recent evidence on the role of LRRK2 suggests that the LRRK2^WD40^ *Drosophila* might represent an optimal PD model for the initial screening of drugs with a disease-modifying potential that acts on the neuroimmune system. LRRK2 is an enzymatic protein involved in multiple cellular functions including neuronal outgrowth, cytoskeletal organization, mitochondrial dynamics, autophagy, protein interactions, and vesicle trafficking (Shin et al., 2008; Cirnaru et al., 2014; Piccoli et al., 2014; Arranz et al., 2015; Roosen and Cookson, 2016). Importantly, more recent reports have shown that LRRK2 is highly expressed in both brain and peripheral immune cells, playing a pivotal role in regulating the neuroinflammatory responses *via* the modulation of nuclear factor (NF)-kB pathway (Gardet et al., 2010; Hakimi et al., 2011; Thevenet et al., 2011; Moehle et al., 2012; Kluss et al., 2019). Notably, NF-kB is central to the efficacious actions of POM in multiple myeloma (Offidani et al., 2014). Moreover, recent evidence points to a pivotal role of LRRK2 in microglia phagocytic function and in *Drosophila* phagocytes ensheathing glia (Kim et al., 2018). As mentioned previously, an unremitting proinflammatory profile together with altered microglia phagocytic function are hallmarks of PD pathogenesis, therefore LRRK2 genetic models are highly appealing for investigating immunomodulatory agents (Joers et al., 2017; Janda et al., 2018).

POM possesses selective and potent TNF-α inhibitory activity, therefore suggesting that the inhibition of inflammatory pathways triggered by the ortholog of this cytokine Eiger in LRRK2^WD40^ flies may be the main neuroprotective mechanism of this agent. Of note, a recent study has provided a clear link between dysfunctional LRRK2 in dopamine neurons and altered glial response in *Drosophila* PD models, by showing that the genetic inhibition of the pro-inflammatory bone morphogenic protein (BMP) signaling in glia, was protective against PD-related neurotoxicity in aged mutant flies (Maksoud et al., 2019). Moreover, although further studies are needed to fully elucidate the functional targets of POM in PD, we suggest that modulation of phagocytosis may represent an additional mechanism of neuroprotection downstream to the anti-inflammatory activity. Hence, phagocytosis is traditionally associated with an anti-inflammatory profile of microglia. In support of this suggestion, we have recently shown in a mouse model of PD that impaired phagocytosis was restored by a neuroprotective agent acting as a modulator of microglia function (Lecca et al., 2018).

In synopsis, our study strongly supports the use of immunomodulatory drugs in PD. Importantly, POM is an FDA-approved clinically available drug, with a toxicological safer profile than the structural analog molecules. If validated in mammalian models of PD, this agent could potentially and rapidly be evaluated in humans.

## Data Availability Statement

The datasets generated for this study are available on request to the corresponding author.

## Author Contributions

MC, IM, MS, and AC conceived, designed the study and analyzed the data. MC, IM, GM, AP, RI, LB, and MS performed the experiments. MC and MS performed the statistical analysis. NG and AC contributed reagents, materials, wrote and revised the manuscript. MC, IM, and MS contributed writing sections of the manuscript. All authors reviewed and approved the submitted version.

## Conflict of Interest

The authors declare that the research was conducted in the absence of any commercial or financial relationships that could be construed as a potential conflict of interest. The reviewer AK declared a past co-authorship with one of the authors MS to the handling Editor.
